# DEMENTIA INTERVENTION STAFF AND CAREGIVER PERSPECTIVES ON THE DELIVERY OF ADS PLUS

**DOI:** 10.1093/geroni/igad104.0524

**Published:** 2023-12-21

**Authors:** Joseph Gaugler, Quinton Cotton, Elizabeth Albers, Stephanie N Ingvalson, Emily Skalla, Laura Gitlin

**Affiliations:** University of Minnesota, Minneapolis, Minnesota, United States; University of Minnesota, Minneapolis, Minnesota, United States; University of Minnesota School of Public Health, Minneapolis, Minnesota, United States; University of Minnesota, Minneapolis, Minnesota, United States; University of Minnesota, Minnesota, Minnesota, United States; Drexel University, Philadelphia, Pennsylvania, United States

## Abstract

Interventions targeting persons living with dementia (PLWDs) provide an optimal touchpoint in the service delivery continuum for addressing the growing unmet needs of unpaid/family dementia caregivers. Services such as Adult Day Services (ADS) that generally target PLWDs can be enhanced by embedding an intervention that specifically targets informal caregivers. In a trial evaluating the ADS Plus intervention, open-ended feedback from ADS intervention staff and participants provided insights to improve the administrative, programmatic, and user experience of ADS Plus. Drawing on individual qualitative interviews (n=24), we conducted a thematic analysis revealing insights into the critical implementation factors supporting the effective delivery of ADS Plus. Identified themes included: 1) the relational, or person-centered, nature of the ADS Plus program, 2) the role of learning throughout implementation, 3) the influence of administrative structure on program delivery, and 4) contributors to the acceptability of ADS Plus. Our findings suggest that caregivers draw on multiple supports but that individually tailored supports for dementia care management must address the emotional needs of caregivers through relational intervention models. The effective delivery of ADS Plus was facilitated by perceived intervention benefits to caregivers, robust yet reasonable training for intervention staff, and the opportunity for adaptability in program delivery.

